# Revised concept of the fossil genus *Oviparosiphum* Shaposhnikov, 1979 with the description of a new genus (Hemiptera, Sternorrhyncha, Aphidomorpha)

**DOI:** 10.3897/zookeys.483.8902

**Published:** 2015-02-19

**Authors:** Dagmara Żyła, Agnieszka Homan, Barbara Franielczyk, Piotr Wegierek

**Affiliations:** 1 Department of Natural History, Upper Silesian Museum, Plac Jana III Sobieskiego 2, 41–902 Bytom, Poland; 2 Department of Zoology, University of Silesia, Bankowa 9, 40–007 Katowice, Poland

**Keywords:** Aphids, fossils, Cretaceous, Oviparosiphidae, *Archeoviparosiphum* gen. n., morphology

## Abstract

This paper presents a revision of the aphid genus *Oviparosiphum*, which is known from the Cretaceous period. Redescriptions of two species: *Oviparosiphum jakovlevi* Shaposhnikov, 1979 and *Oviparosiphum baissense* Shaposhnikov & Wegierek, 1989 are made, and an updated diagnosis of this genus is provided. *Oviparosiphum baissense* is the type species of a newly described genus *Archeoviparosiphum* **gen. n.** Five other species of *Oviparosiphum* are also transferred to the new genus. The basis for their separation from *Oviparosiphum* is the structure of the siphunculi and ovipositor. A key is provided to the genera of Oviparosiphidae.

## Introduction

One aphid family known only from fossils is Oviparosiphidae. Representatives of this family are known from several localities (China, Germany, Mongolia, Russia) but originated within a relatively short time span from the Early/Middle Jurassic to the end of the Early Cretaceous. The oldest genus is *Grimmenaphis* Ansorge, 1996, described from the Lower Jurassic deposit of Grimmen (Germany). However, it is known only from an isolated wing (Ansorge 1996). The oldest undoubted representative is *Khotontaphis* Shaposhnikov & Wegierek, 1989 from the Upper Jurassic/Lower Cretaceous Khotont deposit (Mongolia). More Oviparosiphidae are known from the Early Cretaceous: *Acanthotrichaphis* Shaposhnikov & Wegierek, 1989, *Dinaphis* Shaposhnikov & Wegierek, 1989, *Expansaphis* Hong & Wang, 1990, *Oviparosiphum*, *Sinoviparosiphum* Ren, 1995 and *Vitimaphis* Shaposhnikov & Wegierek, 1989, but they are absent from Upper Cretaceous sediments (Heie and Wegierek 2011).

This family is highly diverse morphologically, but the simultaneous occurrence of the ovipositor and siphunculi constitutes its most characteristic feature (Shaposhnikov 1979). The first described aphid with both these structures visible was *Oviparosiphum jakovlevi* from the Lower Cretaceous Bon–Tsagan deposit (Mongolia), which is the type species of the genus *Oviparosiphum* (Shaposhnikov 1979). To date, the genus includes seven species. The present paper revises the species originally placed in *Oviparosiphum*. It also emends its diagnosis and describes a new genus.

## Material and methods

The material consisted of 44 aphid fossil specimens borrowed from the collection of the Institute of Palaeontology, Russian Academy of Sciences in Moscow. The fossils were preserved in the form of two imprints – “obverse” and “reverse”, marked as ±. The material was analyzed using standard palaeoentomological research methods (Rasnitsyn 2002). Specimens were photographed using a Nikon SMZ1500 stereoscopic microscope and a Nikon Eclipse E600 polarized light microscope. Selected body parts were photographed using Philips XL 30 TMP ESEM and Tescan Vega scanning electron microscopes (with the backscattered electron detector (BSE) in the low–vacuum mode) for better analysis of their morphology. Photographs and measurements were made in the NIS-Elements program. The figures are based on the combined drawings of reverse and obverse imprints, while the photographs represent only one imprint. All measurements are given in mm.

The imprints were collected from two localities: Baissa, Russia and Bon–Tsagaan, Mongolia. Both of them are Lower Cretaceous deposits.

### Geological settings

Baissa is one of the richest deposits and most important localities of fossil insects from the Early Cretaceous. More than 10 000 specimens of insects have been collected from there (Rasnitsyn and Zherikhin 2002). It is located in the Asian part of Russia, in Transbaikalia, in the Buryat Republic, on the left bank of the Vitim River (Kania and Wegierek 2008). The Baissa deposit belongs to the Zaza Formation of approximately Berriasian age (Rasnitsyn et al. 1998). This lithostratigraphic unit is characteristic of the Lower Cretaceous sediments throughout western Transbaikalia. It is built mainly of sandstone, limestone, marl and bituminous shale (Zherikhin et al. 1999).

Bon–Tsagan (= Bon–Tsagaan) is one of the richest Mesozoic insect remains deposits in Mongolia, and one of the best known and richest in the world. Numerous outcrops of mudstone and marls are widely distributed in Central Mongolia, south of the recent Lake Bon–Tsagaan–Nur (Rasnitsyn and Zherikhin 2002). The exact age of the lacustrine sediments of Bon–Tsagaan is estimated at the Early Cretaceous, probably the Aptian. Fossils are well preserved in lacustrine sediments of the lake, which was situated in a mountain valley (Ponomarenko 2013).

## Systematic palaeontology

### Key to the genera of the family Oviparosiphidae:

**Table:** 

1	Less than seven antennal segments	**2**
–	Seven antennal segments	**3**
2	Secondary rhinaria round and irregular	***Expansaphis* Hong & Wang, 1990**
–	Secondary rhinaria annular	***Sinoviparosiphum* Ren, 1995**
3	Siphunculi in the form of short truncate cones	**4**
–	Siphunculi in form of pores	**6**
4	Ovipositor large and well-developed	***Oviparosiphum* Shaposhnikov, 1979**
–	Ovipositor rudimentary	**5**
5	Cubital veins with separate bases	***Vitimaphis* Shaposhnikov & Wegierek, 1989**
–	Cubital veins leave the main vein Sc + R + M from one poin	***Khotontaphis* Shaposhnikov & Wegierek, 1989**
6	Vein CuA_1_ not connected with the main vein Sc + R + M	***Daoaphis* Huang, Wegierek, Żyła & Nel, 2014**
–	Vein CuA_1_ connected with the main vein Sc + R + M	**7**
7	Pterostigma long, at least 5.5 times longer than wide	***Dinaphis* Shaposhnikov & Wegierek, 1989**
–	Pterostigma short, at most 5 times longer than wide	**8**
8	Abdomen with setae	***Acanthotrichaphis* Shaposhnikov & Wegierek, 1989**
–	Abdomen without setae	***Archeoviparosiphum* gen. n.**

### Genus Oviparosiphum

Taxon classificationAnimaliaHemipteraOviparosiphidae

Shaposhnikov, 1979

#### Type species.

*Oviparosiphum jakovlevi* Shaposhnikov, 1979

#### Emended diagnosis.

Seven antennal segments. Secondary rhinaria slightly ellipsoidal, large. Siphunculi in the form of short truncate cones. Ovipositor large with valvae I and III well developed.

### Oviparosiphum jakovlevi

Taxon classificationAnimaliaHemipteraOviparosiphidae

Shaposhnikov, 1979

Figs 1 , 2 , 3 , 4

#### Holotype.

3559/51, Paleontological Institute, Russian Academy of Sciences, Moscow; Bon–Tsagaan locality (Mongolia); Early Cretaceous (Aptian); alate female

#### Emended diagnosis.

As for genus.

#### Redescription.

**Figure 1.**
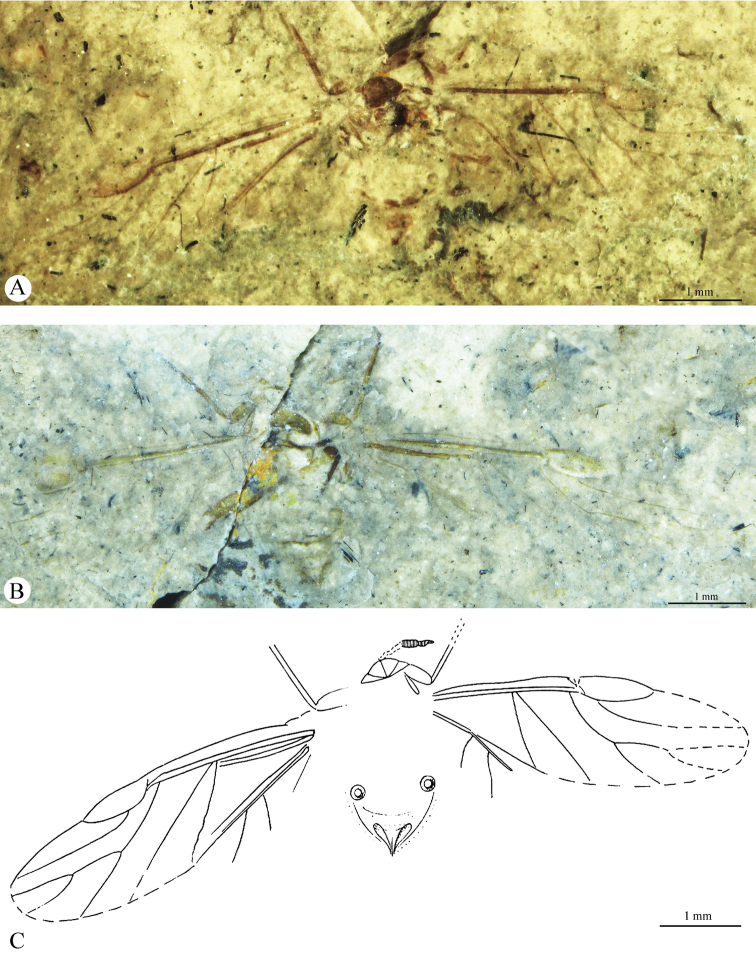
*Oviparosiphum jakovlevi* Shaposhnikov, 1979; PIN 3559/51, holotype. **A** body, ventral view **B** body, dorsal view **C** original reconstruction (after Shaposhnikov 1979).

**Figure 2.**
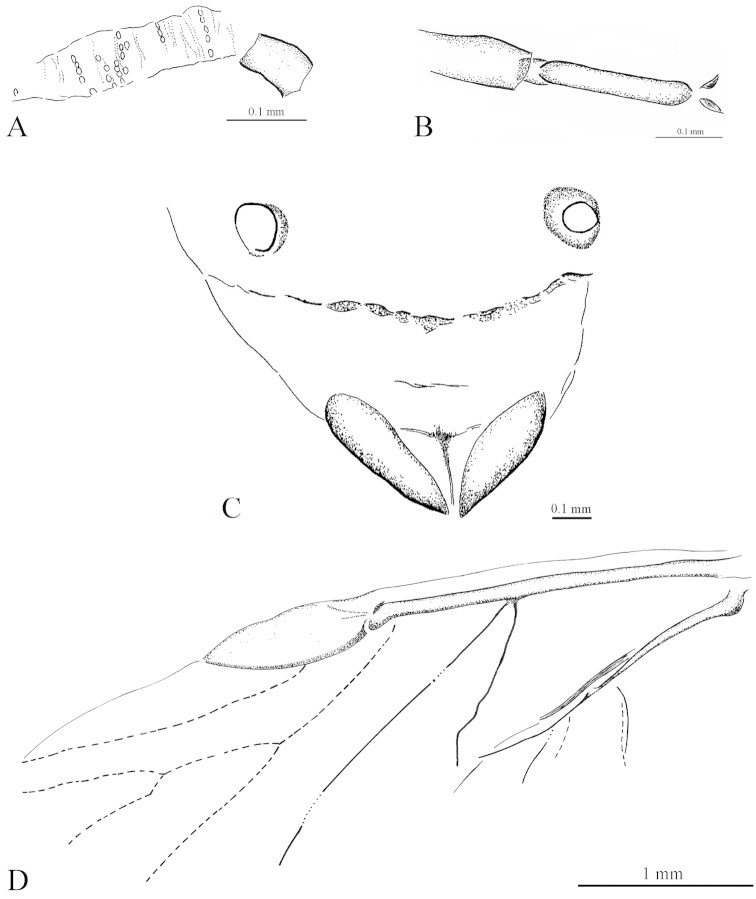
*Oviparosiphum jakovlevi* Shaposhnikov, 1979; PIN 3559/51, holotype. **A** fragment of right antenna with secondary rhinaria, dorsal view **B** middle tarsus **C** apical part of abdomen **D** reconstruction of fore wing.

**Figure 3.**
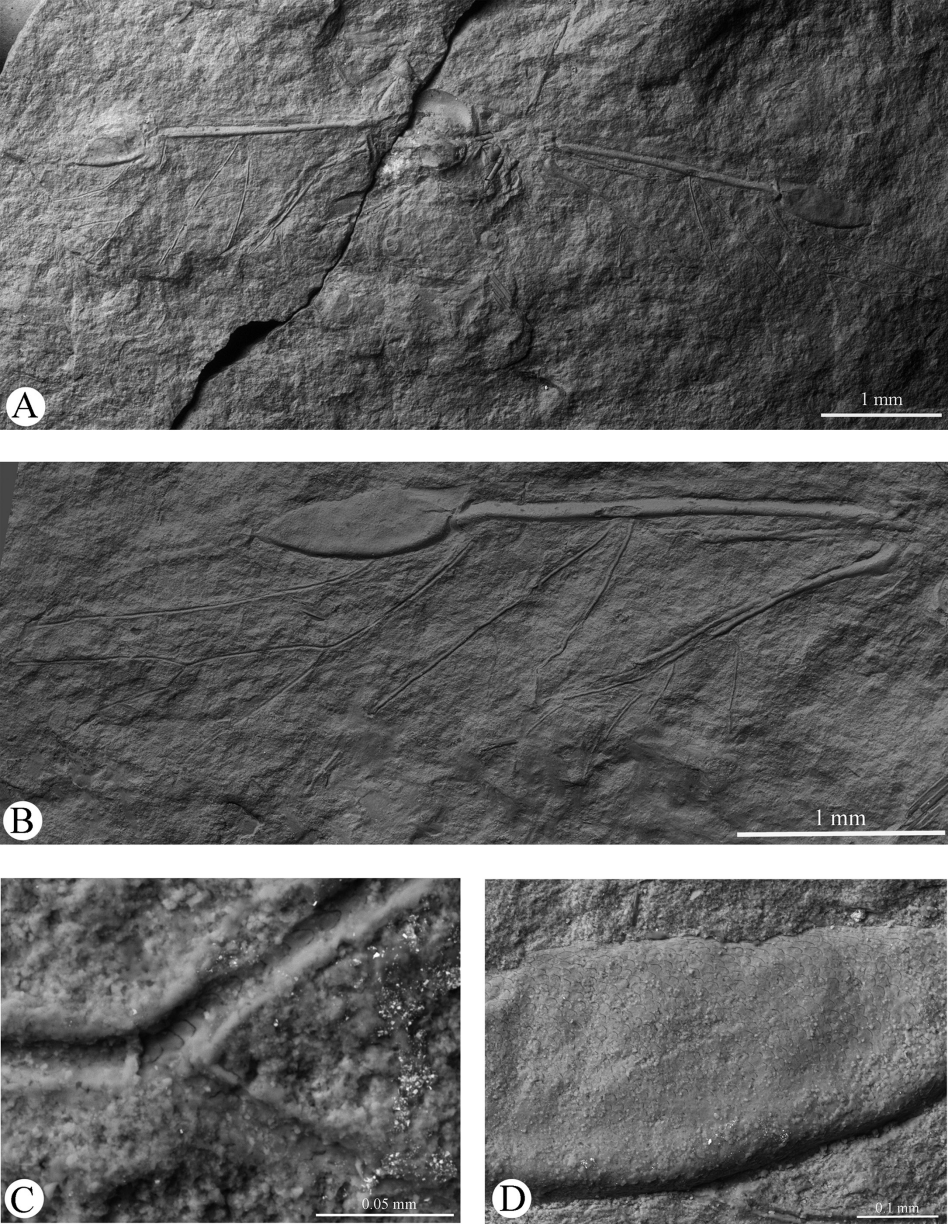
*Oviparosiphum jakovlevi* Shaposhnikov, 1979; PIN 3559/51, holotype, scanning electron micrographs. **A** body, dorsal view **B** fore wing **C** scaly surface of veins **D** scaly surface of pterostigma.

**Figure 4.**
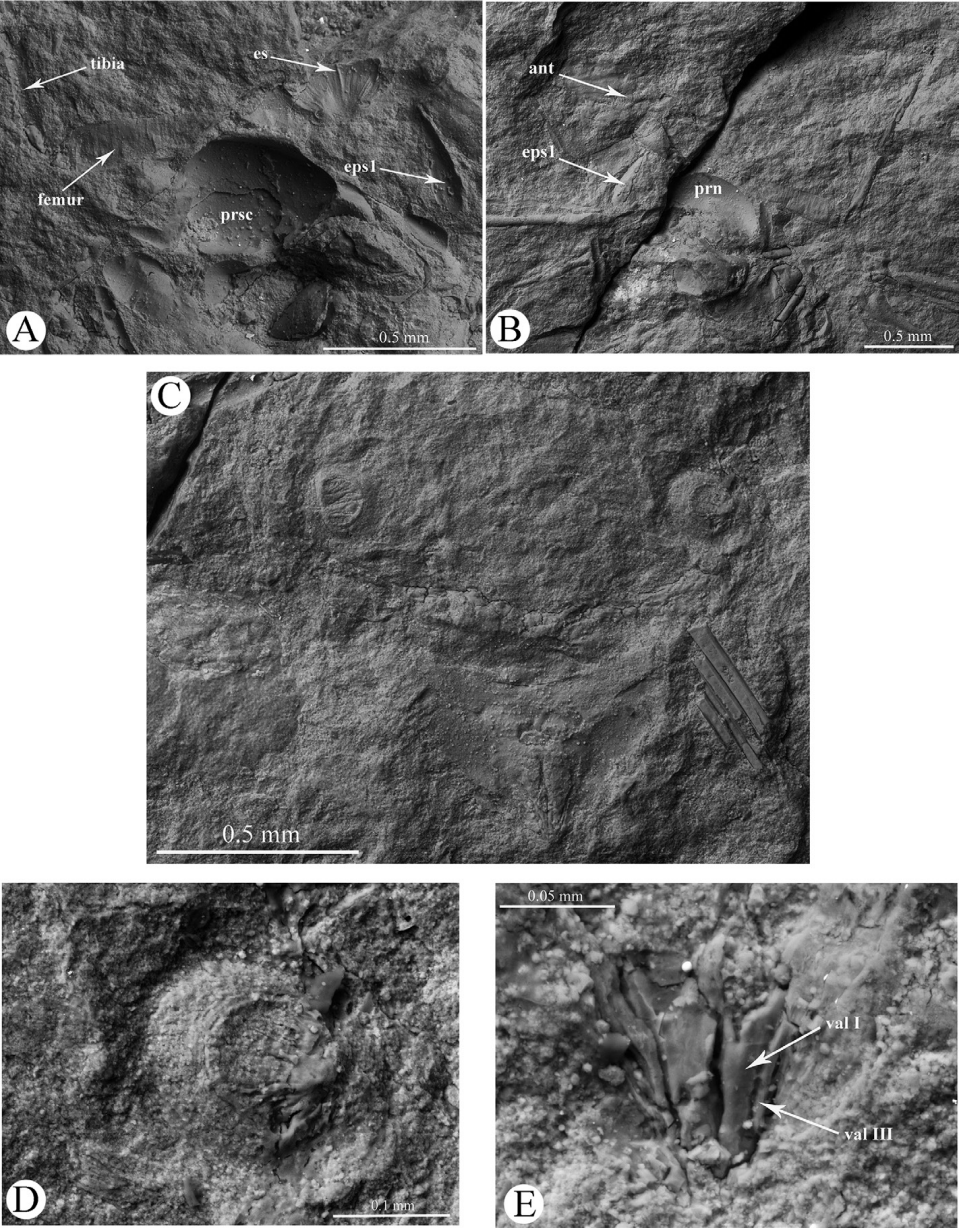
*Oviparosiphum jakovlevi* Shaposhnikov, 1979; PIN 3559/51, holotype, scanning electron micrographs. **A** head and thorax, ventral view **B** head and thorax, dorsal view **C** apical part of abdomen **D** siphunculi; E. ovipositor. ant – antenna, eps1 – proepisternum, es – epicranial suture, prn – pronotum, prsc – praescutum, val – valvae.

Body (2.49) thick (Figs 1A–C, 3A). Epicranial suture present (Fig. 4A), connected in the middle of the epicranium with lateral sutures. On head, narrow oblique slats running from front and lateral edges to the posterior part of the head. Diameter of ocelli 0.05; distance between ocelli 0.24. Segment I of antennae (0.05) shorter than segment II (0.08) (Fig. 4B). Secondary rhinaria arranged in transverse rows (Fig. 2A). Praescutum length 0.37; width 0.52. Femora thick. Fore tibia (1.07) about 2.5 times longer than fore femur (0.38). Length of middle tibia 1.02. Middle tarsus (0.26) (Fig. 2B) about one fourth of middle tibia length. Length of hind coxa 0.11. Hind tibia (1.18) about twice as long as the hind femur (0.53). Hind tibiae about half the body length. Fore wing (4.76) (Figs 2D, 3B) longer than body length. Base of the wing narrow. Vein surface scaly (Figs 3C, D). Distance from base of wing to end of pterostigma 3.33. Bases of cubital veins very close to each other. CuA_1_ (1.46) slightly arched distally, separating from main vein at a 45° angle, slightly shorter than CuA_2_. Vein CuA_2_ (1.16) separating from main vein at mid–length between base of the wing and base of vein Rs, at a 70° angle. Vein M with three branches. Base of vein M directed to base of pterostigma, not connected with main vein. Branches of M form a wide (50°) fork. Base of fork of M_1+2_ and M_3+4_ behind base of vein Rs. Common stem of vein M (0.89) longer than M_1+2_ length (0.63) and equal to M_3+4_ length. Vein Rs (1.67) slightly curved, leaving proximal part of pterostigma at an angle of 25° and running close to it. Pterostigma pointed, short and wide; 3 times longer (0.93) than wide (0.31). Hind wing with two cubital vein. Apical part of abdomen slightly sclerotized (Figs 2C, 4C). Basal diameter of siphunculus 0.13 (Fig. 4D). Diameter of siphunculus aperture 0.10. Ovipositor with valvae I and III well preserved (Fig. 4E). Tergite IX of abdomen clearly visible. Subgenital plate wider than base of ovipositor, 5 times wider than long. In the middle part, its anterior edge forms an indentation reaching half of the length of the plate.

### Genus Archeoviparosiphum gen. n.

Taxon classificationAnimaliaHemipteraOviparosiphidae

http://zoobank.org/B46E2C8B-351B-4392-ADA9-E786234AB825

#### Type species.

*Archeoviparosiphum baissense* (Shaposhnikov & Wegierek, 1989), comb. n.

#### Diagnosis.

Seven antennal segments. Secondary rhinaria slightly ellipsoidal, smaller than secondary rhinaria of *Oviparosiphum*. Vein CuA_1_ connected with the main vein Sc + R + M. Pterostigma short, at most 5 times longer than wide. Abdomen without setae. Siphunculi in form of pores. Ovipositor small and rudimentary.

#### Etymology.

The name is a combination of the Greek word *archaios* (meaning ‘ancient’) and the genus *Oviparosiphum*.

#### Gender.

Neuter.

### Archeoviparosiphum baissense

Taxon classificationAnimaliaHemipteraOviparosiphidae

(Shaposhnikov & Wegierek, 1989) comb. n.

Figs 5 , 6

#### Holotype.

4210/2623±, Paleontological Institute, Russian Academy of Sciences, Moscow; Baissa locality (Russia); Early Cretaceous; alate female

#### Paratypes.

3064/2107; 3064/2108(2273); 3064/2161(2164); 3064/2165; 3064/2166; 3064/2171(5138); 3064/2181; 3064/2184; 3064/2193(2210); 3064/2203; 3064/2205; 3064/2213±; 3064/2309(2311); 3064/2310(2312); 3064/2313(4846); 3064/2314; 3064/2315(2316); 3064/3978; 3064/4765; 3064/4775; 3064/4816; 3064/4911; 3064/4915; 3064/4916(4922); 3064/4970; 3064/4975; 3064/5031; 3064/5110; 4210/2521±; 4210/2624±; 4210/2625; 4210/2771; 4210/2801±; 4210/2802; 4210/2803

#### Additional material.

1989/1072; 3064/2160; 3064/2173(4751); 3064/2225; 3064/2276; 3064/2278; 3064/2284; 3064/4825; 3064/4870; 3064/4990; 3064/5023; 3064/5113; 3064/5130a; 4210/2548; 4210/4120±; 4210/4365±; 4210/4629±; 4210/5553±; 4210/5588±; 4210/5590±; 4210/5618±; 4210/5630±; 4210/5654; 4210/5659; 4210/5661; 4210/5665; 4210/5669; 4210/5674; 4210/5675; 4210/6889±; 4210/6893±; 4210/6894±; 4210/6896±; 4210/6899±; 4210/6900±; 4210/6908; 4210/6911; 4210/6912a; 4210/6915; 4210/7577a±; 4210/7577b±; 4210/7580±; 4210/7584±

#### Emended diagnosis.

Bases of cubital veins leaving the main vein at the same point.

#### Description.

**Figure 5.**
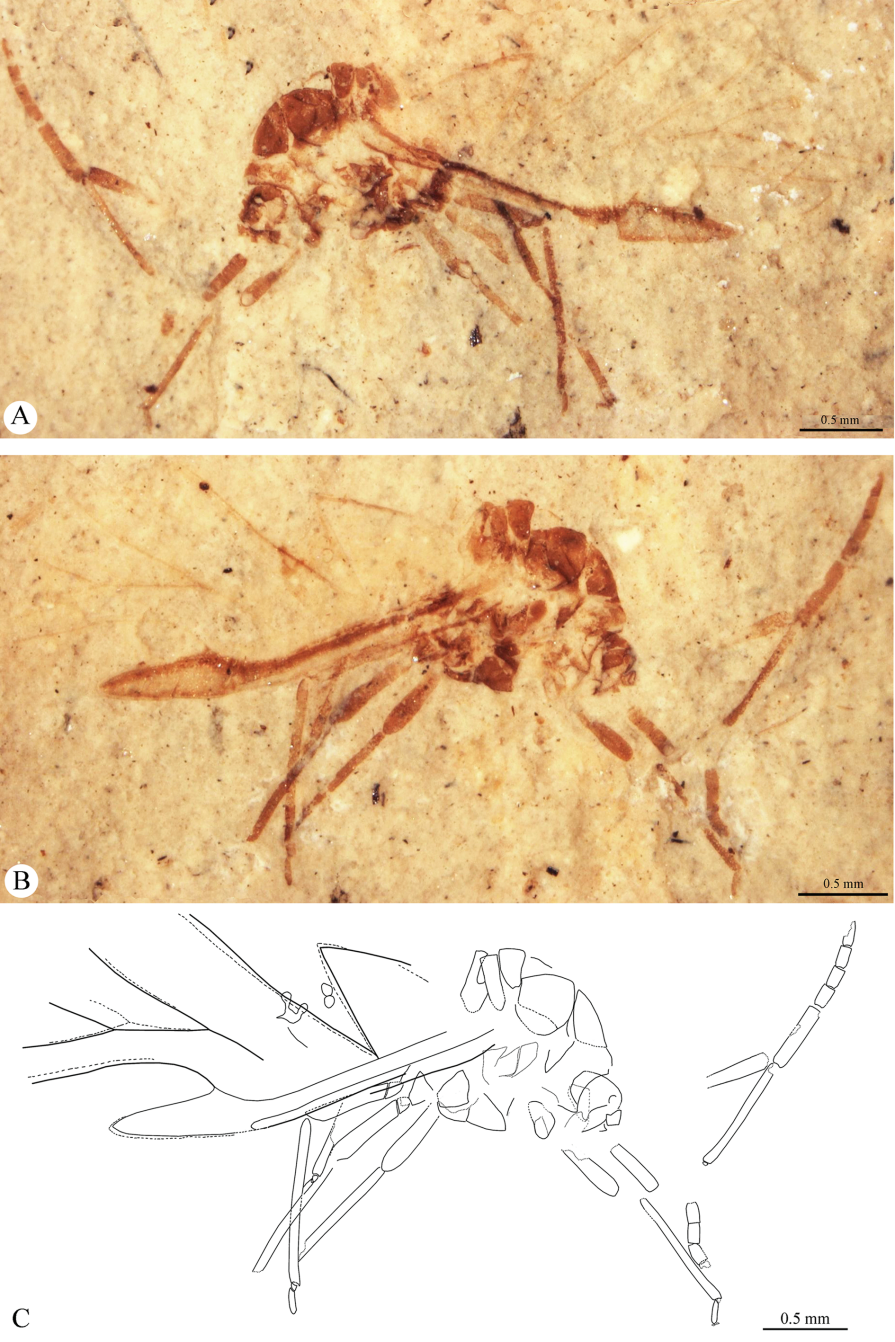
*Archeoviparosiphum baissense* (Shaposhnikov & Wegierek, 1989), comb. n.; PIN 4210/2623±, holotype. **A, B** body, lateral views **C** reconstruction of body.

**Figure 6.**
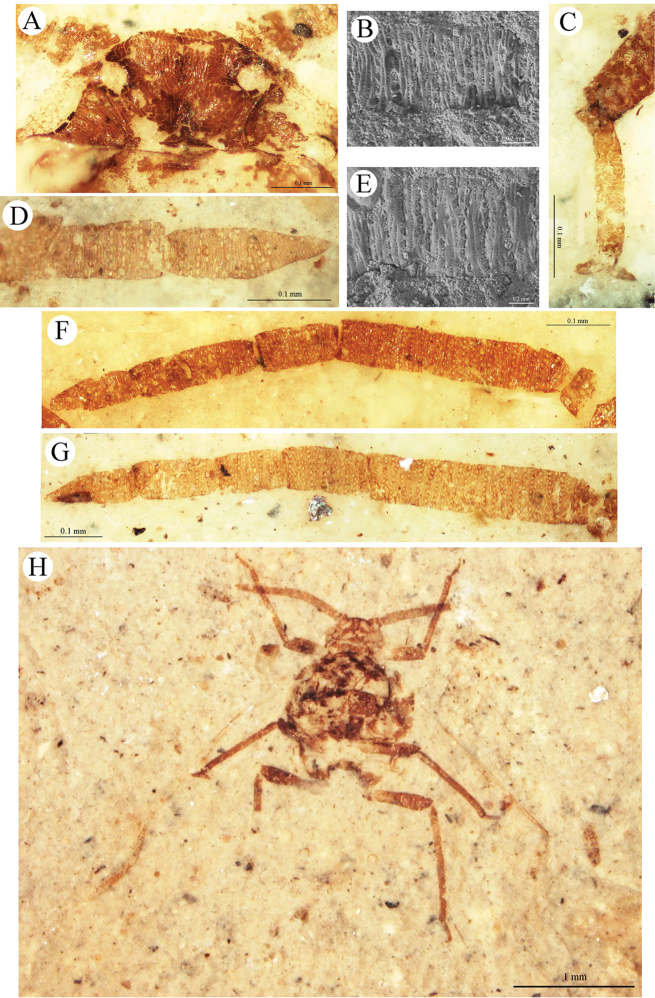
*Archeoviparosiphum baissense* (Shaposhnikov & Wegierek, 1989), comb. n. **A** PIN 3064/2213±, paratype, head, dorsal view **B** PIN 3064/2284, additional material, fragment of antennal segment V with secondary rhinaria, scanning electron micrograph **C** PIN 3064/2310(2312), paratype, hind tarsus **D** PIN 3064/2203, paratype, antennal segment VI and VII with secondary rhinaria **E** PIN 3064/2284, additional material, fragment of antennal segment III with secondary rhinaria, scanning electron micrograph **F** PIN 3064/2108(2273), paratype, right antenna **G** PIN 3064/2310(2312), paratype, left antenna **H** PIN 3064/2213±, paratype, body, ventral view.

Body (1.9–2.7) thick (Figs 5A–C, 6H). Anterior margin of head (Fig. 6A) straight. Length of the head (0.19–0.25); width (0.35–0.40). Epicranial suture present, sometimes not clearly visible. Lateral sutures present. On head, additional narrow oblique slats running from front and lateral edges to the posterior part of the head. Distance between ocelli 0.18–0.20. Antennae (0.94–1.40) (Figs 6F, G) half of body length, and longer or equal to hind tibiae length. Segments I 0.05–0.06 and II 0.04–0.06 in length. Segment III (0.34–0.42) shorter than the cumulative length of subsequent segments; about 3–4 times longer than wide. Segment IV (0.11–0.14) as long as wide, equal to segment V and VI. Segment VII (0.15–0.18) slightly longer than the previous segment, about twice as long as wide, narrowed in the apical part. Secondary rhinaria arranged in dense, transverse rows (Figs 6B, D, E), on segment III in 24–30 rows; segments IV–VI in 7–10 rows; segment VII in 5–8 rows. 8–11 secondary rhinaria in one row on segment III. Rostrum relatively long, reaching to the hind coxae. Length of mesoscutellum 0.13–0.15; width 0.39–0.45. Length of mesothoracic sternite 0.41–0.46; width 0.68–0.72. Length of fore femur 0.34; fore tibia 0.61–0.71; segment I of fore tarsus 0.03; segment II 0.13–0.14. Length of middle femur 0.39–0.49; middle tibia 0.68–0.78; segment I of middle tarsus 0.03; segment II 0.15–0.17. Length of hind coxa 0.15–0.16; hind trochanter 0.10; hind femur 0.48–0.53; hind tibia 0.89–1.28; segment I of hind tarsus 0.04 (Fig. 6C); segment II 0.17–0.19. Hind tibiae about half the body length. Fore wing (3.10–3.60) longer than body length. Distance from base of wing to end of pterostigma 2.50–2.70. Vein CuA_1_ about twice as long as CuA_2_. Vein M three branched, its base directed to base of pterostigma, not connected with main vein. M furcates to M_1+2_ and M_3+4_ behind Rs base. Common stem of vein M longer than M_1+2_. Vein Rs slightly curved, leaving proximal part of the pterostigma and running far away from it. Pterostigma pointed, long and narrow; 4 times longer than wide. Hind wing (2.70) with two cubital veins. Apical part of abdomen slightly sclerotized. Diameter of siphunculus 0.07–0.08.

## Discussion

The first species of the genus *Oviparosiphum*, *Oviparosiphum jakovlevi* was described in 1979 by Shaposhnikov and assigned to a new family Oviparosiphidae. The diagnostic features were the following: antennae with annular secondary rhinaria, siphunculi in the form of truncated cones with a height less than the diameter of the wide aperture, and a large conical ovipositor, most likely composed of four valvae (Shaposhnikov 1979). New techniques made a detailed study possible, confirming the presence of these features and allowing to redescribe the type species of the genus *Oviparosiphum*. Additionally, analysis of the antennal morphology showed that the secondary rhinaria of *Oviparosiphum jakovlevi* are slightly ellipsoidal and occur in dense, transverse rows. The second species assigned to this genus was *Oviparosiphum baissense* from the Baissa deposit (Lower Cretaceous, Russia), described on the basis of a single imprint (Shaposhnikov and Wegierek 1989). The large number of specimens available for the present study permitted a very accurate redescription of the species. The presence of pore-shaped siphunculi has been clearly demonstrated, in contrast to the original description, which highlighted the short cone-shaped siphunculi. The characteristics that distinguish these two species of the genus *Oviparosiphum* from other genera in the family are: similar length of both cubital veins and vein Rs leaving proximal part of the pterostigma. These features remain valid but more attention is paid to the abdomen structure. Only one other genus – *Khotonaphis* – has clearly truncate conical siphunculi, but its ovipositor is not as well developed as in *Oviparosiphum jakovlevi* (Shaposhnikov and Wegierek 1989). The species *Oviparosiphum latum*, described from the Early Cretaceous of China, is more problematic because vein Rs leaves the distal part of the pterostigma, and the fact that the exact structure of the secondary rhinaria is unknown (Hong and Wang 1990). However, the drawings suggest that the siphunculi are pore-shaped, which enables it to be reclassified as a member of the new genus.

In the original description of *Paroviparosiphum* syn. n. and *Mesoviparosiphum* syn. n. the authors indicated 5-segmented antennae, annular secondary rhinaria and pore-shaped siphunculi as being diagnostic features of both genera (Zhang et al. 1989). However, on the basis of drawings, it could be stated that the antennae are 7-segmented, typical of the Oviparosiphidae, and that the secondary rhinaria are most likely ellipsoidal. Nevertheless the siphunculi seem to be pore-shaped, which makes it possible to include these genera also in *Archeoviparosiphum*.

The composition of the new genus is thus as follows:

***Archeoviparosiphum baissense*** (Shaposhnikov & Wegierek, 1989), comb. n.

*Oviparosiphum baissensis* Shaposhnikov & Wegierek, 1989: 49 (original combination)

***Archeoviparosiphum camptotropum*** (Zhang, Zhang, Hou & Ma, 1989), comb. n.

*Paroviparosiphum camptotropum* Zhang, Zhang, Hou & Ma, 1989: 31 (original combination)

*Oviparosiphum camptotropum* (Zhang, Zhang, Hou & Ma, 1989) (synonym by Heie and Wegierek 2011: 49)

***Archeoviparosiphum latum*** (Hong & Wang, 1990), comb. n.

*Oviparosiphum latum* Hong & Wang, 1990: 80 (original combination)

***Archeoviparosiphum malacum*** (Zhang, Zhang, Hou & Ma, 1989), comb. n.

*Mesoviparosiphum malacum* Zhang, Zhang, Hou & Ma, 1989: 33 (original combination)

*Oviparosiphum malacum* (Zhang, Zhang, Hou & Ma, 1989) (synonym by Heie and Wegierek 2011: 49)

***Archeoviparosiphum opimum*** (Zhang, Zhang, Hou & Ma, 1989), comb. n.

*Paroviparosiphum opimum* Zhang, Zhang, Hou & Ma, 1989: 29 (original combination)

*Oviparosiphum opimum* (Zhang, Zhang, Hou & Ma, 1989) (synonym by Heie and Wegierek 2011: 49)

***Archeoviparosiphum tuanwangense*** (Zhang, Zhang, Hou & Ma, 1989), comb. n.

*Mesoviparosiphum tuanwangense* Zhang, Zhang, Hou & Ma, 1989: 32 (original combination)

*Oviparosiphum tuanwangense* (Zhang, Zhang, Hou & Ma, 1989) (synonym by Heie and Wegierek 2011: 49)

## Conclusion

Previously the genus *Oviparosiphum* consisted of seven species. It is now limited to a single species, *Oviparosiphum jakovlevi*, with clearly truncate conical siphunculi and a well–developed ovipositor. The other species have been transferred to a new genus *Archeoviparosiphum* gen. n.; all these species have pore-shaped siphunculi and a rudimentary ovipositor.

## Supplementary Material

XML Treatment for Oviparosiphum

XML Treatment for Oviparosiphum jakovlevi

XML Treatment for Archeoviparosiphum

XML Treatment for Archeoviparosiphum baissense
